# Diastolic left ventricular function in relation to the retinal microvascular fractal dimension in a Flemish population

**DOI:** 10.1038/s41440-021-00623-3

**Published:** 2021-02-04

**Authors:** Fang-Fei Wei, Lutgarde Thijs, Jesus D. Melgarejo, Nicholas Cauwenberghs, Zhen-Yu Zhang, Chen Liu, Tatiana Kuznetsova, Harry A. J. Struijker-Boudier, Peter Verhamme, Yu-Gang Dong, Jan A. Staessen

**Affiliations:** 1 Studies Coordinating Centre, Research Unit Hypertension and Cardiovascular Epidemiology, Department of Cardiovascular Sciences, University of Leuven, Leuven, Belgium; 2 Department of Cardiology, The First Affiliated Hospital of Sun Yat-sen University, Guangzhou, China; 3 Department of Pharmacology, Maastricht University, Maastricht, The Netherlands; 4 Centre for Molecular and Vascular Biology, Department of Cardiovascular Sciences, University of Leuven, Leuven, Belgium; 5 Research Institute Alliance for the Promotion of Preventive Medicine, Mechelen, Belgium

**Keywords:** Fractal dimension, Left ventricular diastolic function, Population science, Retinal microcirculation

## Abstract

Fractal analysis provides a global assessment of vascular networks (e.g., geometric complexity). We examined the association of diastolic left ventricular (LV) function with the retinal microvascular fractal dimension. A lower fractal dimension signifies a sparser retinal microvascular network. In 628 randomly recruited Flemish individuals (51.3% women; mean age, 50.8 years), we measured diastolic LV function by echocardiography and the retinal microvascular fractal dimension by the box-counting method (Singapore I Vessel Assessment software, version 3.6). The left atrial volume index (LAVI), e′, E/e′ and retinal microvascular fractal dimension averaged (±SD) 24.3 ± 6.2 mL/m^2^, 10.9 ± 3.6 cm/s, 6.96 ± 2.2, and 1.39 ± 0.05, respectively. The LAVI, E, e′ and E/e′ were associated (*P* < 0.001) with the retinal microvascular fractal dimension with association sizes (per 1 SD), amounting to −1.49 mL/m^2^ (95% confidence interval, −1.98 to −1.01), 2.57 cm/s (1.31–3.84), 1.34 cm/s (1.07–1.60), and −0.74 (−0.91 to −0.57), respectively. With adjustments applied for potential covariables, the associations of E peak and E/e′ with the retinal microvascular fractal dimension remained significant (*P* ≤ 0.020). Over a median follow-up of 5.3 years, 18 deaths occurred. The crude and adjusted hazard ratios expressing the risk of all-cause mortality associated with a 1-SD increment in the retinal microvascular fractal dimension were 0.36 (0.23–0.57; *P* < 0.001) and 0.57 (0.34–0.96; *P* = 0.035), respectively. In the general population, a lower retinal microvascular fractal dimension was associated with greater E/e′, a measure of LV filling pressure. These observations can potentially be translated into new strategies for the prevention of diastolic LV dysfunction.

## Introduction

Heart failure (HF) is a major global health problem [1] and the leading cause of morbidity and mortality worldwide [2]. Approximately 50% of HF patients present with symptoms of diastolic dysfunction with preserved ejection fraction [3]. Subclinical diastolic left ventricular (LV) dysfunction has a prevalence of 25% in the general population [4, 5], predisposes patients to further deterioration of LV function [6], and finally progresses to overt HF [3].

A novel paradigm of diastolic HF focuses on proinflammatory signaling originating from the myocardial microvasculature [7]. However, noninvasively phenotyping the myocardial microvasculature in populations is not practicable. In contrast, the retinal microvasculature can be completely noninvasively [8] and reproducibly [9] assessed. Furthermore, fractal analysis of the retinal microvasculature provides a global assessment of the complexity and density of small vessels [10]. A lower fractal dimension signifies a sparser retinal microvascular network. The retinal microvascular fractal dimension predicts long-term microvascular complications in patients with diabetes [11] and the incidence of coronary mortality in the general population [12]. Along these lines, the retinal microvasculature might provide a window representative of the microcirculation throughout the human body, including the myocardium. To test the hypothesis that diastolic LV function might be related to microcirculatory dysfunction [7], we examined the association of echocardiographically assessed diastolic LV function with the retinal microvascular fractal dimension in a Flemish population study [13].

## Methods

### Study population

The Flemish Study on Environment and Genes in Relation to Health Outcomes (FLEMENGHO) complies with the Helsinki declaration for research in humans [14]. The Ethics Committee of the University Hospitals Leuven approved the protocol. FLEMENGHO participants were recruited from a geographically defined area in northern Belgium. Enrollment of FLEMENGHO participants started in 1985. From August 1985 until November 1990, a random sample of households living in a geographically defined area of northern Belgium was investigated with the goal of recruiting an equal number of participants in each of six subgroups by sex and age (20–39, 40–59, and ≥60 years). All household members with a minimum age of 20 years were invited to take part, given that the quota of their sex-age group had not yet been satisfied. From June 1996 until January 2004, recruitment of families continued using the former participants (1985−1990) as index persons and including teenagers [15]. The initial participation rate was 78.0%. The participants were repeatedly followed up. In all study phases, we used the same standardized methods to assess the clinical characteristics of the participants, to administer questionnaires and to ascertain the incidence of adverse health outcomes. At each contact, participants gave or renewed informed written consent. From 2005 until 2014, the re-examination included echocardiography (re-examination rate, 80.3%). Of 964 participants, we excluded 43 subjects because of atrial fibrillation (*n* = 10) or paced heart rhythm (*n* = 3) or because of insufficient quality of the echocardiographic recordings (*n* = 30). Of 921 participants, 631 had both gradable echocardiographic and retinal images available for analysis. We additionally excluded three participants because the left atrial volume index or retinal microvascular fractal dimension was more than three SDs away from the population mean. Thus, the number of participants statistically analyzed totaled 628.

### Retinal photography

Participants were asked to refrain from heavy exercise, smoking, and drinking alcohol or caffeine-containing beverages for at least 3 h prior to being examined. We applied a nonmydriatic approach in a dimly lit room to acquire retinal photographs, one image per eye in each participant, with the Canon Cr-DGi retinal visualization system and the Canon D 50 digital camera (Canon Inc., Medical Equipment Group, Utsunomiya, Japan). After centering the image on the optic disk as shown in Supplementary Fig. 1, we measured a range of retinal microvascular traits using the validated computer-assisted program SIVA (Singapore I Vessel Assessment, version 3.6, Singapore Eye Research Institute, Singapore). The retinal vascular fractal dimension quantifies the complexity of the branching pattern of the retinal vascular tree and is computed using a box-counting method, with larger values indicating a more complex branching pattern. It is derived from the gradient between the logarithm of the number of boxes (log n) plotted against the box size (log *ε*) [16]. For the retinal microvascular fractal dimension, the intraobserver Qi-Fang Huang (Q-FH) variability expressed as a percentage of the average value and the intraclass correlation coefficient were 1.4% and 0.99 [17]. The corresponding interobserver (F-FW and Q-FH) estimates were 2.3% and 0.95 [17].

### Echocardiography

Echocardiographic measurements and retinal photographs were obtained on the same examination day. Echocardiographic images were acquired and analyzed off-line according to current guidelines [18]. Previous publications have described the procedures applied for acquisition and the off-line analysis of the echocardiographic measurements in detail. In short, echocardiographic images were obtained with a Vivid7 Pro device (GE Vingmed, Horten, Norway) interfaced with a 2.5–3.5 MHz phased-array probe. For off-line analysis, we applied EchoPac software, version 4.0.4 (GE Vingmed, Horten, Norway) and averaged the measurements over three heart cycles. Left atrial volume was calculated using the prolate ellipsoid method. We determined the peak early (E) and peak late (A) diastolic velocities of the transmitral blood flow from the pulsed Doppler signal as well as the peak early (e′) and peak late (a′) velocities of the mitral annular movement by tissue Doppler imaging (TDI), with velocities averaged over four acquisition sites (septal, lateral, inferior, and posterior). The intraobserver reproducibility of the single observer in the study, defined as the 2-SD interval about the mean of the relative differences between duplicate readings, across the four TDI sampling sites, ranged from 4.5% to 5.3% for e′ and from 4.0% to 4.5% for a′ [6].

### Ascertainment of mortality

At annual intervals, we ascertained the vital status of all participants via the National Population Registry (Brussels, Belgium). The cause of death was ascertained by record linkage with the Flemish Registry of Death Certificates. Linkage was achieved with permission of the Belgian Data Protection Authority (https://www.dataprotectionauthority.be).

### Statistical analysis

For database management and statistical analysis, we used SAS software, version 9.4 (SAS Institute Inc., Cary, NC, USA). We compared means and proportions by the large sample z-test or ANOVA and by the *χ*^2^-statistic, respectively. The central tendency (spread) was represented by the arithmetic mean (SD) for normally distributed variables and by the geometric mean (interquartile range) of logarithmically transformed variables. Statistical significance was a two-sided α-level of 0.05.

In exploratory analyses, we determined the differences in echocardiographic measurements across thirds of the retinal microvascular fractal dimension distribution. We applied mixed models to model the association of LV traits with the retinal microvascular fractal dimension while accounting for clustering within families (random effect). We expressed the differences in the echocardiographic measurements in relation to the retinal microvascular fractal dimension per 1-SD increment. In multivariable-adjusted analyses, in line with previous publications [4, 13], we accounted for sex, age, body mass index, mean arterial pressure, heart rate, total cholesterol, plasma glucose, γ-glutamyltransferase as an index of alcohol intake, smoking, antihypertensive drug treatment by class, and history of cardiovascular disease. The left atrial volume index was standardized to body surface area and was therefore not adjusted for body mass index.

In an attempt to validate our observations, we applied proportional hazard regression to assess the risk of all-cause mortality as a function of the retinal microvascular dimension at baseline stratified by the median of the distribution. To account for confounding, we computed a propensity score defined as the retinal microvascular fractal dimension predicted by other covariables, including sex, age, body mass index, mean arterial pressure, heart rate, total cholesterol, plasma glucose, γ-glutamyltransferase, smoking, antihypertensive drug treatment by class, and history of cardiovascular disease.

## Results

### Characteristics of participants

All 628 participants were white Europeans, of whom 322 (51.3%) were women. Among all participants, the mean values (±SDs) were 50.8 ± 14.6 years for age, 26.8 ± 4.4 kg/m^2^ for body mass index, and 129.0 ± 16.4/81.7 ± 9.9 mmHg for systolic/diastolic blood pressure. The prevalence of hypertension and diabetes mellitus was 41.1% and 2.9%, respectively. Of 258 participants with hypertension, 143 were on antihypertensive drug treatment with β-blockers (*n* = 79 [55.2%]), inhibitors of the renin-angiotensin system (angiotensin-converting enzyme inhibitors or angiotensin II type-1 receptor blockers; *n* = 53 [37.1%]), vasodilators (calcium-channel blockers or α-blockers; *n* = 37 [25.9%]) or diuretics (*n* = 54 [37.8%]), prescribed in varying combinations in 63 (44.0%) patients. Eighteen (2.9%) participants had diabetes mellitus. The mean values (±SDs) of the left atrial volume index, E and e′ peak, E/e′ and retinal microvascular fractal dimension were 24.3 ± 6.2 mL/m^2^, 70.8 ± 16.0 cm/s, 10.9 ± 3.6 cm/s, 6.96 ± 2.2, and 1.39 ± 0.05, respectively. Supplementary Fig. 2 shows the distributions of the echocardiographic measurements, and Supplementary Fig. 3 shows the distribution of the retinal microvascular fractal dimension.

Table 1 lists the characteristics of the participants by thirds of the retinal microvascular fractal dimension distribution. Across increasing categories (Table 1) of age, body mass index, blood pressure, and plasma glucose, the prevalence of hypertension, diabetes mellitus, and a history of cardiovascular disease decreased (*P* ≤ 0.026).

**Table 1:** Characteristics of participants by thirds of the fractal dimension distribution

Characteristics	Category of fractal dimension			*P* value
Limits	≤1.372	1.372−1.415	>1.415	
Number of participants (%)	209 (33.3)	210 (33.4)	209 (33.3)	
All participants in category				
Women	97 (46.4)	116 (55.2)	109 (52.2)	0.186
Current smoking	30 (14.4)	35 (16.7)	40 (19.1)	0.423
Drinking alcohol	83 (39.7)	87 (41.4)	73 (34.9)	0.367
Hypertension	122 (58.4)	88 (41.9)^‡^	48 (23.0)^‡^	<0.001
Antihypertensive treatment				
Diuretics	33 (15.8)	16 (7.6)^†^	5 (2.4)*	<0.001
β-Blockers	40 (19.1)	25 (11.9)*	14 (6.7)	0.001
ACEIs or ARBs	38 (18.2)	11 (5.2)^‡^	4 (1.9)	<0.001
CCBs or α-blockers	23 (11.0)	12 (5.7)*	2 (1.0)^†^	<0.001
Diabetes mellitus	12 (5.7)	5 (2.4)	1 (0.5)	0.005
History of CVD	16 (7.7)	3 (1.4)^†^	3 (1.4)	<0.001
Mean of characteristic				
Age (years)	58.2 ± 14.3	50.0 ± 13.1^‡^	44.3 ± 12.9^‡^	<0.001
Body mass index (kg/m2)	27.3 ± 4.3	26.8 ± 4.6	26.2 ± 4.3	0.026
Systolic pressure (mmHg)	135.8 ± 17.6	128.0 ± 14.3^‡^	123.1 ± 14.6^†^	<0.001
Diastolic pressure (mmHg)	82.6 ± 9.5	83.2 ± 9.7	79.2 ± 10.1^‡^	<0.001
Total cholesterol (mmol/L)	5.06 ± 0.96	5.10 ± 0.91	4.98 ± 0.89	0.350
γ-glutamyltransferase (U/L)	30.0 (9.7–70.8)	22.6 (6.4–49.4)	17.0 (4.7–48.3)	0.008
Plasma glucose (mmol/L)	5.04 ± 0.80	4.82 ± 0.65^‡^	4.73 ± 0.39	<0.001

For continuously distributed characteristics, the arithmetic mean (±SD) was given and the geometric mean (interquartile range) for the logarithmically transformed distribution of γ-glutamyltransferase. Body mass index was body weight in kilogram divided by height in meter squared. Hypertension was a blood pressure (average of five consecutive readings) of ≥140 mmHg systolic or ≥90 mmHg diastolic or use of antihypertensive drugs. Diabetes mellitus was fasting plasma glucose of ≥126 mg/dL (7.0 mmol/L) or use of antidiabetic agents

*ACEIs* angiotensin-converting enzyme inhibitors, *ARBs* angiotensin-receptor blockers, *CCBs* calcium-channel blockers, *CVD* cardiovascular disease

*P* values are for the difference in prevalence (*χ*^2^ test) or mean (ANOVA) across categories of retinal microvascular fractal dimension. Significance of the difference with the adjacent lower category: *P ≤ 0.05; ^†^*P* ≤ 0.01; ^‡^*P* ≤ 0.001

### Diastolic LV function in relation to fractal dimension

Table 2 lists the echocardiographic and retinal traits by thirds of the retinal microvascular fractal dimension. The left atrial volume index, the A and a′ peaks, and the E/e′ ratio decreased (*P* < 0.001) with a higher category of the retinal microvascular fractal dimension, whereas the E and e′ peaks, the E/A and e′/a′ ratios, and the central retinal arteriolar and venular diameters increased from the low to high category (*P* ≤ 0.001). Supplementary Table 1 shows the echocardiographic and retinal microvascular characteristics of the participants by sex. Compared with men, women had a smaller left atrial volume index and a′ peak but had higher transmitral A and E peak velocities, E/e′ ratio values, and central retinal arteriolar and venular diameters (*P* ≤ 0.042).

**Table 2:** Echocardiographic and retinal traits by thirds of retinal microvascular fractal dimension distribution

Characteristics	Category of fractal dimension			*P* value
Limits	≤1.372	1.372−1.415	>1.415	
Number of participants (%)	209 (33.3)	210 (33.4)	209 (33.3)	
Echocardiographic				
LAVI (mL/m^2^)	26.2 ± 6.9	23.7 ± 5.9^‡^	23.1 ± 5.3	<0.001
A peak (cm/s)	65.5 ± 16.2	58.7 ± 14.4^‡^	53.6 ± 12.5^‡^	<0.001
E peak (cm/s)	67.9 ± 16.3	70.6 ± 16.0	73.9 ± 15.2*	0.001
E/A ratio	1.12 ± 0.45	1.29 ± 0.49^‡^	1.46 ± 0.49^‡^	<0.001
e′ peak (cm/s)	9.34 ± 3.4	11.0 ± 3.3^‡^	12.4 ± 3.3^‡^	<0.001
a′ peak (cm/s)	9.91 ± 2.2	9.34 ± 2.0^†^	8.75 ± 1.9^†^	<0.001
e′/a′ ratio	1.05 ± 0.62	1.30 ± 0.64^‡^	1.57 ± 0.73^‡^	<0.001
E/e′ ratio	7.88 ± 2.8	6.77 ± 1.8^‡^	6.22 ± 1.5^†^	<0.001
Retinal				
CRAE (µm)	153.1 ± 13.2	155.4 ± 12.2*	159.2 ± 11.5^†^	<0.001
CRVE (µm)	222.1 ± 18.8	228.2 ± 17.6^‡^	231.9 ± 16.2*	<0.001
AVR	0.69 ± 0.05	0.68 ± 0.05	0.69 ± 0.05	0.178
Fractal dimension	1.33 ± 0.03	1.39 ± 0.01^‡^	1.44 ± 0.02^‡^	<0.001

*LAVI* left atrial volume index, *CRAE* central retinal arteriolar equivalent, *CRVE* central retinal venular equivalent, *AVR* arteriole-to-venule diameter ratio

*P* values are for the difference in means (ANOVA) across categories of retinal microvascular fractal dimension. Significance of the difference with the adjacent lower category: *P ≤ 0.05; ^†^*P* ≤ 0.01; ^‡^*P* ≤ 0.001

**Fig. 1:**
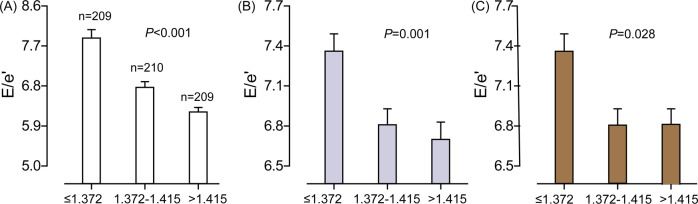
E/e′ ratio by thirds of the retinal microvascular fractal dimension distribution in unadjusted analyses (**A**), in analyses adjusted for sex and age (**B**), and in analyses additionally adjusted for body mass index, mean arterial pressure, heart rate, total cholesterol, plasma glucose, γ-glutamyltransferase as an index of alcohol intake, smoking, antihypertensive drug treatment by class, and history of cardiovascular disease (**C**). The *P* value is for the trend. Bars indicate the SE

In exploratory analyses, the E/e′ ratio decreased across increasing categories of the retinal microvascular dimension, irrespective of whether E/e′ was analyzed as a continuous outcome (Fig. 1) or was dichotomized by the median of the distribution (Supplementary Fig. 4). In analyses that accounted for clustering within families but otherwise were unadjusted (Table 3), the associations with the retinal microvascular fractal dimension were inverse for the left atrial volume index and the E/e′ ratio and were positive for the E and e′ peaks; the association sizes were −1.49 mL/m^2^, −0.74, 2.57 cm/s, and 1.34 cm/s, respectively. With adjustments applied as described in the statistical methods, a 1-SD increment in the retinal microvascular fractal dimension was associated with a lower E/e′ ratio (−0.19; *P* = 0.013; Table 3).

**Table 3:** Association of left ventricular diastolic function with retinal microvascular fractal dimension

Echocardiographic traits	Unadjusted models		Adjusted models	
	Estimate (95% CI)	*P* value	Estimate (95% CI)	*P* value
LAVI (mL/m^2^)	−1.49 (−1.98 to −1.01)	<0.001	0.01 (−0.45 to 0.47)	0.964
E peak (cm/s)	2.57 (1.31–3.84)	<0.001	−1.42 (−2.61 to −0.23)	0.020
e′ peak (cm/s)	1.34 (1.07–1.60)	<0.001	−0.06 (−0.23–0.10)	0.446
E/e′ ratio	−0.74 (−0.91 to −0.57)	<0.001	−0.19 (−0.35 to −0.04)	0.013

Association sizes (95% confidence interval) express the difference in the echocardiographic traits per 1-SD increment in the retinal microvascular fractal dimension. All estimates accounted for clustering within families. Adjusted models accounted for sex, age, body mass index, mean arterial pressure (diastolic blood pressure plus one-third of pulse pressure), heart rate, total cholesterol, plasma glucose, γ-glutamyltransferase as an index of alcohol consumption, smoking, antihypertensive drug treatment by class, and history of cardiovascular disease. LAVI was standardized to body surface area and was therefore not adjusted for body mass index.

*LAVI* left atrial volume index

### Total mortality and retinal fractal dimension

**Fig. 2:**
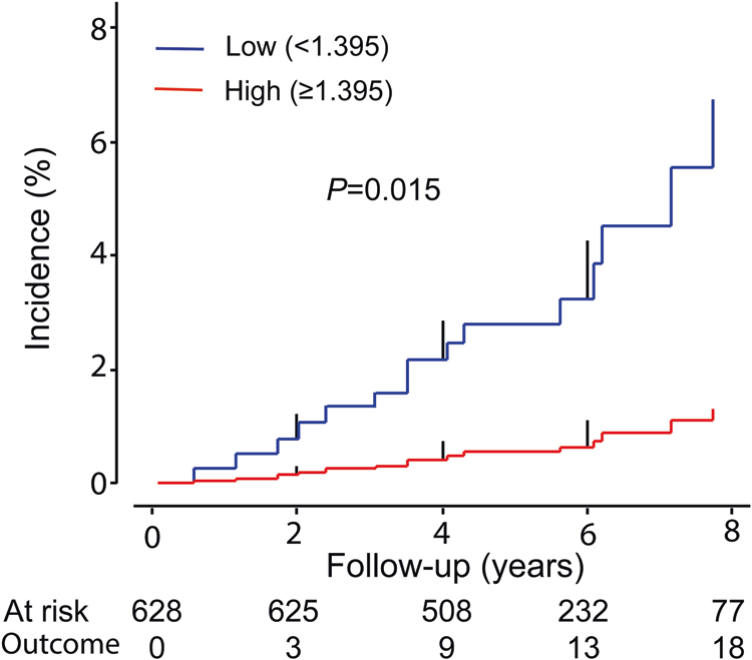
Cumulative incidence of all-cause mortality by the median retinal microvascular fractal dimension at baseline in 628 participants. The mean levels of the retinal microvascular fractal dimension (interquartile range) in the low and high groups were 1.350 (1.329–1.378) and 1.430 (1.409–1.445), respectively. The *P* value is for the between-group difference. Vertical lines denote the SE. The median follow-up was 5.3 years

Over a median follow-up of 5.3 years (5th–95th percentile interval, 3.2–8.7 years), 18 deaths occurred, of which 3 (16.7%) were cardiovascular deaths. HF was not the immediate cause of death in any of the patients but was a contributory cause in two patients. Compared with the high median group of retinal fractal dimension distribution, the low median group had a higher incidence of all-cause mortality (Fig. 2). The hazard ratio expressing the crude risk of all-cause mortality associated with a 1-SD increment in the retinal microvascular fractal dimension was 0.36 (95% confidence interval [CI], 0.23–0.57; *P* < 0.001). In a model adjusted for the propensity score, the hazard ratio was 0.57 (0.34–0.96; *P* = 0.035). In receiver operating characteristic plots (Supplementary Fig. 5), adding the retinal microvascular fractal dimension to the E/e′ ratio increased (*P* = 0.048) the area under the curve from 0.74 (0.61–0.87) to 0.83 (0.72–0.94).

## Discussion

Assuming that the retinal microvasculature might provide a window representative of the myocardial microcirculation, the key finding of our study was that parameters of diastolic function, i.e., the Doppler-derived mitral inflow velocity (E peak) and mitral annular early diastolic velocity (E/e′ ratio), were inversely associated with the retinal microvascular fractal dimension. This association persisted after adjustment for sex, age, body mass index, mean arterial pressure, and other potential confounders.

The retinal microvascular fractal dimension is a mathematical measure that quantifies complex geometric patterns of the retinal microvascular network [10]. A lower fractal dimension signifies a sparser retinal microvascular network. Previous studies have demonstrated that the retinal microvascular fractal dimension is associated with hypertension [19], diabetic retinopathy [20], chronic kidney disease [21], stroke [22], and coronary heart disease [12]. In a case-control study of 557 patients with ischemic stroke and 557 controls, the retinal microvascular fractal dimension was lower (1.379 vs. 1.421; *P* < 0.001) in cases than in controls [22]. In a population study of 3303 adults (age, ≥49 years), the multivariable-adjusted hazard ratio of coronary mortality (*n* = 468) in the lowest fourth of the distribution of the retinal microvascular fractal dimension relative to medium-low and medium-high fourths was 1.51 (95% CI 1.17–1.94; *P* = 0.002) [12]. Furthermore, in 180 type-1 diabetic patients followed for 16 years, a lower retinal microvascular dimension predicted incident neuropathy (adjusted odds ratio per 0.01 fractal dimension decrease, 1.17; 95% CI, 1.01–1.36) and nephropathy (adjusted odds ratio, 1.40; 95% CI, 1.10–1.79), supporting our assumption that the retinal microvasculature is representative of the microvasculature in distant organs, particularly in the peripheral nervous system and the kidney in this study. Along similar lines, the E/e′ ratio, a measure of diastolic LV filling pressure, confers risk, as demonstrated in 816 high-risk hypertensive patients enrolled in the Anglo-Scandinavian Cardiac Outcomes Trial [23]. Over 4.2 years of follow-up (mean), 56 cardiac endpoints occurred. The multivariable-adjusted hazard ratio for a 1-unit increment in E/e′ was 1.17 (95% CI, 1.05–1.29; *P* = 0.003) [23]. Other studies confirmed the risk associated with the E/e′ ratio in diabetic patients [24] and in the general population [25].

Two observations provide validation of our key results. First, the echocardiographic and retinal microvascular traits showed the established sex differences. Compared with men, women had a smaller left atrial volume index and a′ peak but had higher transmitral A and E peak velocities, E/e′ ratio values, and central retinal arteriolar and venular diameters. Second, total mortality over a median follow-up of 5.3 years was substantially lower with a greater retinal microvascular fractal dimension as measured at the time of echocardiographic and retinal phenotyping. The hazard ratio was 0.36 (95% CI, 0.23–0.57; *P* < 0.001). This observation was consistent with the adjustment for propensity score. Similar to other investigators [26], we chose this modality of adjustment in view of the low number of fatal endpoints. The strong points of our study also include the population-based design, which is less prone to bias than observations in patient cohorts; the application of a validated computer-assisted technique for the off-line analysis of the retinal microvasculature [27]; and the high intra- and interobserver reproducibility in reading the retinal traits [17]. However, our study also has potential limitations, including its cross-sectional design, which precludes direct causal inferences, and its generalizability to populations with a different lifestyle, those living under different environmental conditions, or those belonging to other ethnicities. Of 921 participants, 290 (31.5%) had no gradable echocardiographic or retinal images and were therefore excluded from the current analysis. However, compared with those 290 subjects, participants with both gradable echocardiographic and retinal images available for analysis had similar (*P* ≤ 0.072) sex distribution, body mass index, diastolic blood pressure, and prevalence of smoking and alcohol intake but were on average 5.2 years older (*P* < 0.001) and, therefore, had a slightly higher (+5.7 mmHg, *P* < 0.001) systolic blood pressure. FLEMENGHO is a population-based study. The prevalence of asymptomatic diastolic LV dysfunction was approximately 27% [4]. Guideline-proposed thresholds for the E/e′ ratio were derived in symptomatic patients [28] but are not applicable to asymptomatic people randomly recruited from the population. We constructed thresholds for the echocardiographic indexes of diastolic left ventricle dysfunction in the general population using age-specific criteria derived from a normal reference group nested within FLEMENGHO [4]. These thresholds were reproduced in a randomly recruited Polish population sample [5]. Our current study only included patients with subclinical diastolic left LV dysfunction, but no patients had overt HF. This precludes any inference with regard to systolic or diastolic heart failure.

In Flemish individuals randomly recruited from the general population, a lower retinal microvascular fractal dimension was associated with greater E/e′, a measure of LV filling pressure. These observations can potentially be translated into new strategies for prevention in individuals at risk of diastolic LV dysfunction. In randomly recruited European population samples, the frequency of asymptomatic echocardiographically diagnosed diastolic LV dysfunction was as high as 25% [4, 5], with a 10% risk of progression to HF over 5 years [6]. Once HF was diagnosed, over 40% of patients died within 1 year of their first hospitalization, and 25% were readmitted within 1 year [29]. Assessment of the retinal microvasculature may help to provide the means to stratify HF risk and to initiate preventive measures in a timely manner, long before irreversible cardiac damage sets in. According to current guidelines, prevention should focus on managing the risk factors for LV dysfunction [30], such as hypertension, obesity, dyslipidemia, progressive renal dysfunction, and insulin resistance.

## Supplementary information

Data Supplement

## References

[1] Bui AL, Horwich TB, Fonarow GC (2011). Epidemiology and risk profile of heart failure. Nat Rev Cardiol.

[2] Cook C, Cole G, Asaria P, Jabbour R, Francis DP (2014). The annual global economic burden of heart failure. Int J Cardiol.

[3] Paulus WJ, Tschöpe C (2013). A novel paradigm for heart failure with preserved ejection fraction. J Am Coll Cardiol.

[4] Kuznetsova T, Herbots L, López B, Jin Y, Richart T, Thijs L (2009). Prevalence of left ventricular diastolic dysfunction in a general population. Circ Heart Fail.

[5] Kloch-Badelek M, Kuznetsova T, Sakiewicz W, Tikhonoff V, Ryabikov A, González A, et al. Prevalence of diastolic left ventricular dysfunction in European populations based on cross-validated diagnostic thresholds. Cardiovasc Ultrasound. 2012;10. 10.1186/1476-7120-10-10. 10.1186/1476-7120-10-10 PMC3351014 22429658

[6] Sharrett AR, Hubbard LD, Cooper LS, Sorlie PD, Brothers RJ, Nieto FJ (1999). Retinal arteriolar diameters and elevated blood pressure. The Atherosclerosis Risk in Communities Study. Am J Epidemiol.

[7] Nieuwdorp M, Holleman F, de Groot E, Vink H, Gort J, Kontush A (2007). Perturbation of hyaluronan metabolism predisposes patients with type 1 diabetes mellitus to atherosclerosis. Diabetologia.

[8] Liew G, Wang JJ, Mitchell P, Wong TY (2008). Retinal vascular imaging: a new tool in microvascular disease research. Circ Cardiovasc Imaging.

[9] Wei FF, Zhang ZY, Petit T, Cauwenberghs N, Gu YM, Thijs L (2016). Retinal microvascular diameter, a hypertension-related trait, in ECG-gated vs. non-gated images analyzed by IVAN and SIVA. Hypertens Res.

[10] Patton N, Aslam TM, MacGillivray T, Deary IJ, Dhillon B, Eikelboom RH (2006). Retinal image analysis: concepts, applications and potential. Prog Retin Eye Res.

[11] Broe R, Rasmussen ML, Frydkjaer-Olsen U, Olsen BS, Mortensen HB, Peto T (2014). Retinal vascular fractals predict long-term microvascular complications in type 1 diabetes mellitus: the Danish Cohort of Pediatric Diabetes 1987 (DCPD1987). Diabetologia.

[12] Liew G, Mitchell P, Rochtchina E, Wong TY, Hsu W, Lee ML (2011). Fractal analysis of retinal microvasculature and coronary heart disease mortality. Eur Heart J.

[13] Kuznetsova T, Thijs L, Knez J, Cauwenberghs N, Petit T, Gu YM (2015). Longitudinal changes in left ventricular diastolic function in a general population. Circ Cardiovasc Imaging.

[14] World Medical Association. (2013). World Medical Association Declaration of Helsinki. Ethical principles for medical research involving human subjects. J Am Med Ass.

[15] Yang WY, Petit T, Thijs L, Zhang ZY, Jacobs L, Hara A (2015). Coronary risk in relation to genetic variation in MEOX2 and TCF15 in a Flemish population. BMC Genet.

[16] Huang F, Dashtbozorg B, Zhang J, Bekkers E, Abbasi-Sureshjani S, Berendschot TTJM (2016). Reliability of using retinal fractal dimensions as a biomarker in the diabetic retinopathy detection. J Ophtalmol.

[17] Huang QF, Wei FF, Zhang ZY, Raaijmakers A, Asayama K, Thijs L (2017). Reproducibility of retinal microvasculaar traits decoded by the Singapore I Vessel Assessment Software across the human age range. Am J Hypertens.

[18] Nagueh SF, Smiseth OA, Appleton CP, Byrd BF3, Dokainish H, Edvardsen T (2016). Recommendations for the evaluation of left ventricular diastolic function by echocardiography: an update from the American Society of Echocardiography and the European Association of Cardiovascular Imaging. J Am Soc Echocardiogr.

[19] Liew G, Wang JJ, Cheung N, Zhang YP, Hsu W, Lee ML (2008). The retinal vasculature as a fractal: methodology, reliability, and relationship to blood pressure. Ophthalmology.

[20] Grauslund J, Green A, Kawasaki R, Hodgson L, Sjølie AK, Wong TY (2010). Retinal vascular fractals and microvascular and macrovascular complications in type 1 diabetes. Ophthalmology.

[21] Sng CC, Sabanayagam C, Lamoureux EL, Liu E, Lim SC, Hamzah H (2010). Fractal analysis of the retinal vasculature and chronic kidney disease. Nephrol Dial Transpl.

[22] Ong YT, De Silva DA, Cheung CY, Chang HM, Chen CP, Wong MC (2013). Microvascular structure and network in the retina of patients with ischemic stroke. Stroke.

[23] Sharp ASP, Tapp RJ, McG Thom SA, Francis DP, Hughes AD, Stanton AV (2010). Tissue Doppler E/E’ ratio is a powerful predictor of primary cardiac events in a hypertensive population: an ASCOT substudy. Eur Heart J.

[24] Blomstrand P, Engvall M, Festin K, Lindstrõm T, Länne T, Maret E (2015). Left ventricular diastolic function, assessed by echocardiography and tissue Doppler imaging, is a strong predictor of cardiovascular events, superior to global left ventricular longitudinal strain, in patients with type 2 diabetes. Eur Heart J Cardiovascular Imaging.

[25] Redfield MM, Jacobsen SJ, Burnett JC, Mahoney DW, Bailey KR, Rodeheffer RJ (2003). Burden of systolic and diastolic ventricular dysfunction in the community: appreciating the scope of the heart failure epidemic. J Am Med Ass.

[26] Halley CM, Houghtaling PL, Khalil MK, Thomas JD, Jaber WA (2011). Mortality rate in patients with diastolic dysfunction and normal systolic function. Arch Intern Med.

[27] Wang JJ, Liew G, Klein R, Rochtchina E, Knudtson MD, Klein BEK (2007). Retinal vessel diameter and cardiovascular mortality: pooled data analysis from two older populations. Eur Heart J.

[28] Yancy CW, Jessup M, Bozkurt B, Butler J, Casey DE, Drazner MH, Fonarow GC, Geraci SA, Horwich T, Januzzi JL, Johnson MR, Kasper EK, Levy WC, Masoudi FA, McBride PE, McMurray JJV, Mitchell JE, Peterson PN, Riegel B, Sam F, Stevenson LW, Tang WHW, Tsai EJ, Wilkoff BL (2013). ACCF/AHA guideline for the management of heart failure: a report of the American College of Cardiology Foundation/American Heart Association Task Force on Practice Guidelines. J Am Coll Cardiol.

[29] Wang H, Dwyer-Lindgren L, Lofgren KT, Rajaratnam JK, Marcus JR, Levin-Rector A (2012). Age-specific and sex-specific mortality in 187 countries, 1970–2010: a systematic analysis for the Global Burden of Disease Study 2010. Lancet.

[30] Prasad SB, Guppy-Coles KB, Holland D, Stanton T, Krishnasamy R, Whalley G (2019). Echocardiographic predictors of all-cause mortality in patients with left ventricular ejection fraction >35%: value of guideline based assessment of diastolic dysfunction. Int Cardiol Heart Vasc.

