# Supplementary material for: Diastolic left ventricular function in relation to the retinal microvascular fractal dimension in a Flemish population
Source: Hypertens Res. 2021 Feb 4;44(4):446–53. doi: 10.1038/s41440-021-00623-3 (PMC8019655; doi:10.1038/s41440-021-00623-3)
Supplement: Supplementary file 1 — Data Supplement [file 41440_2021_623_MOESM1_ESM.doc]

***Hypertension Research***

**Data Supplement**

**Diastolic left ventricular function in relation to
retinal microvascular fractal dimension in a Flemish population**

Fang-Fei Wei, Lutgarde Thijs, Jesus D. Melgarejo, Nicholas Cauwenberghs,
Zhen-Yu Zhang, Chen Liu, Tatiana Kuznetsova, Harry A.J. Struijker-Boudier,
Peter Verhamme, Yu-Gang Dong, Jan A. Staessen*

* Correspondence to: Dr Jan A. Staessen, Studies Coordinating Centre, Research Unit Hypertension and Cardiovascular Epidemiology, KU Leuven Department of Cardiovascular Sciences, Campus Sint Rafaël, University of Leuven, Kapucijnenvoer 35, Box 7001, BE-3000 Leuven, Belgium; Email: [jan.staessen@med.kuleuven.be](mailto:jan.staessen@med.kuleuven.be).

**Table of Contents**

| Supplementary Table 1 | Echocardiographic and Retinal Measurements by sex | p2 |
| --- | --- | --- |
| Supplementary Figure 1 | The retinal microvasculature as visualized by nonmydriatic photography | p3 |
| Supplementary Figure 2 | Frequency distributions of left atrial volume index (LAVI), E peak, e’ peak and E/e’ ratio | p4 |
| Supplementary Figure 3 | Frequency distribution of retinal microvascular fractal dimension | p5 |
| Supplementary Figure 4 | Prevalence of low and high E/e’ ratio (≤6.5 and >6.5, respectively) by thirds of the distribution of retinal microvascular fractal dimension | p6 |
| Supplementary Figure 5 | Receiver operating characteristics plots discriminates the area under the curve between E/e’ ratio (red line) and E/e’ ratio plus retinal microvascular fractal dimension (blue line) | p7 |

**Supplementary Table 1**

Echocardiographic and Retinal Measurements by Sex.

| Characteristic | Men (N=306) | Women (N=322) | All (N=628) |
| --- | --- | --- | --- |
| Echocardiographic |  |  |  |
| LA volume index (mL/m^2^) | 25.7±6.6 | 23.0±5.6‡ | 24.3±6.2 |
| A peak (cm/s) | 56.0±14.5 | 62.3±15.3‡ | 59.2±15.2 |
| E peak (cm/s) | 67.0±15.2 | 74.4±16.0‡ | 70.8±16.0 |
| E/A ratio | 1.29±0.50 | 1.29±0.50 | 1.29±0.50 |
| e' peak (cm/s) | 10.8±3.6 | 11.0±3.5 | 10.9±3.6 |
| a' peak (cm/s） | 9.61±2.1 | 9.07±2.1† | 9.33±2.1 |
| e'/a' ratio | 1.26±0.69 | 1.35±0.70 | 1.31±0.70 |
| E/e' ratio | 6.61±1.9 | 7.29±2.4‡ | 6.96±2.2 |
| Retinal arterioles |  |  |  |
| CRAE (µm) | 154.2±12.5 | 157.5±12.4‡ | 155.9±12.5 |
| Simple tortuosity | 1.10±0.03 | 1.10±0.03 | 1.10±0.03 |
| Curvature tortuosity (x10-5) | 4.98±1.3 | 4.93±1.1 | 4.95±1.2 |
| Branching angle (°) | 82.4±12.5 | 82.8±11.1 | 82.6±11.8 |
| Asymmetry factor | 0.81±0.09 | 0.81±0.08 | 0.81±0.09 |
| Retinal venules |  |  |  |
| CRVE (µm) | 225.9±18.0 | 228.8±18.0* | 227.4±18.0 |
| Simple tortuosity | 1.10±0.01 | 1.10±0.02 | 1.10±0.02 |
| Curvature tortuosity (x10-5) | 5.20±0.95 | 5.19±1.1 | 5.19±1.1 |
| Branching angle (°) | 82.1±8.7 | 82.1±7.9 | 82.1±8.3 |
| Asymmetry factor | 0.75±0.09 | 0.72±0.08 | 0.72±0.08 |
| AVR | 0.68±0.05 | 0.69±0.05 | 0.69±0.05 |
| Fractal dimension | 1.39±0.05 | 1.39±0.05 | 1.39±0.05 |

Abbreviations: AVR, arteriole-to-venule diameter ratio; CRAE, central retinal arteriolar diameter; CRVE, central retinal venular diameter; LA, left atrial. Values are means±SD. *P* values indicate significance of the differences between sex: * *P*≤0.05; † *P*≤0.01; ‡ *P*≤0.01.


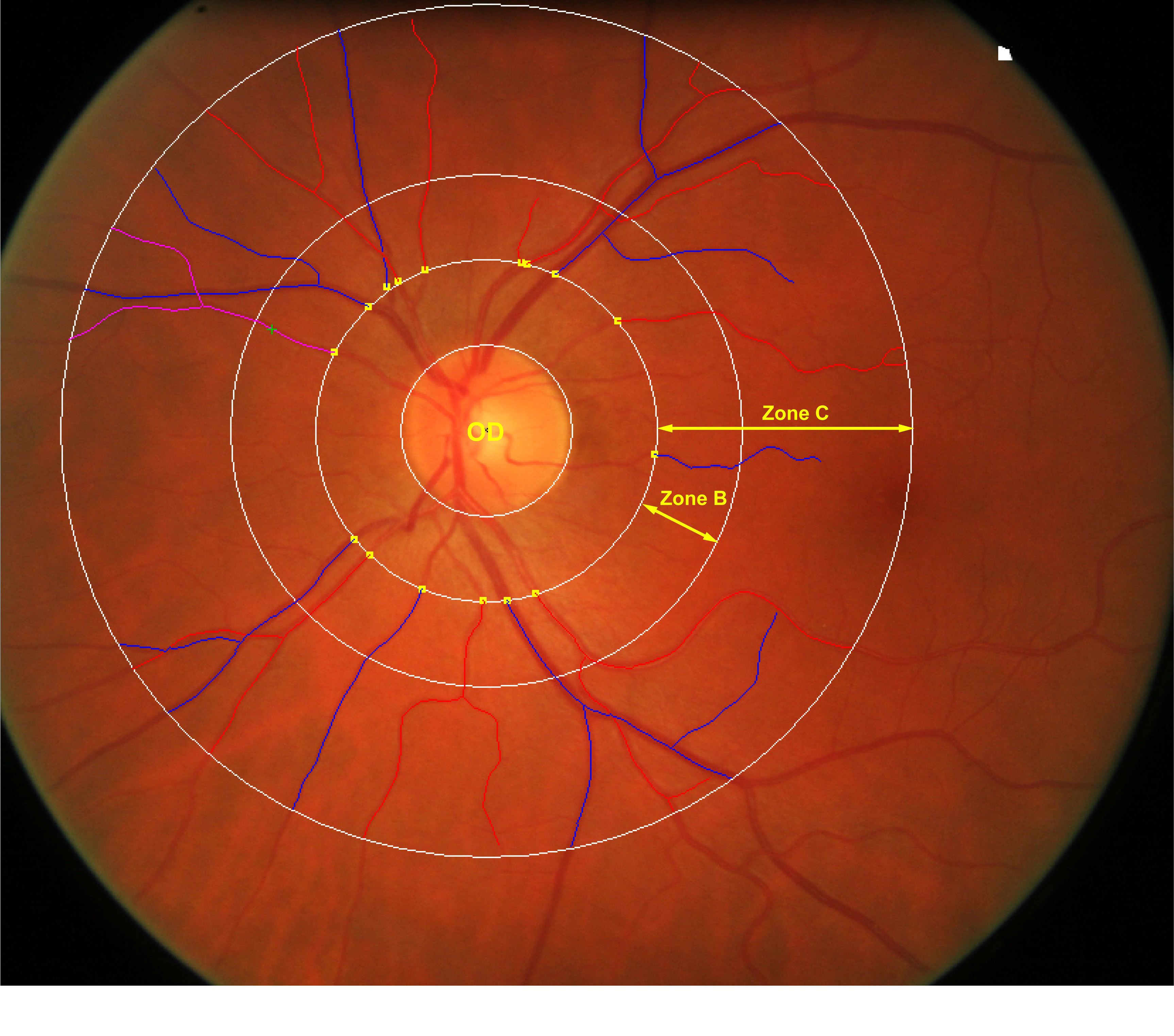


**Supplementary Figure 1**

The retinal microvasculature as visualized by nonmydriatic photography. Arterioles are represented in red and venules in blue. The retinal microvascular traits, including the arteriolar and venular calibers and the fractal dimensions were measured using the validated computer assisted program SIVA (Singapore I Vessel Assessment, version 3.6, Singapore Eye Research Institute, Singapore) in zones B and C, which are respectively 0.5 to 2.0 disc diameters away of the optic disc margin.


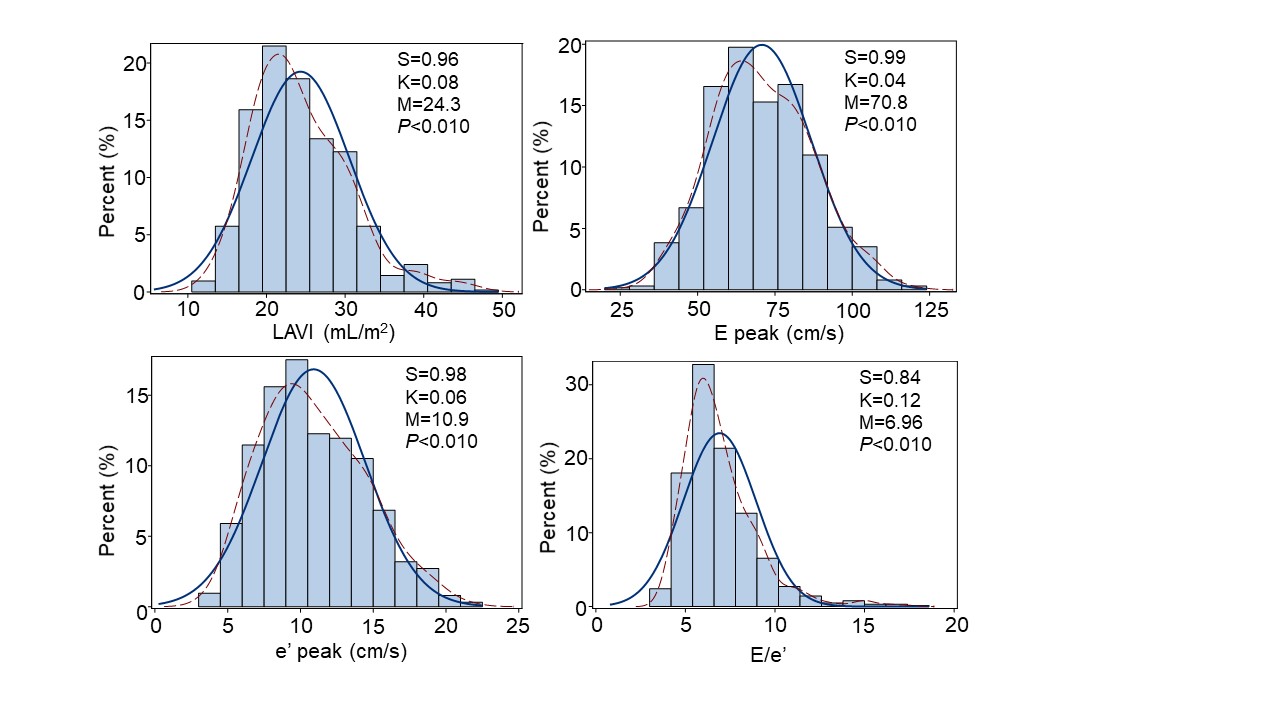


**Supplementary Figure 2**

Frequency distributions of left atrial volume index (LAVI), E peak, e’ peak and E/e’ ratio. S, K and M are the coefficients of skewness and kurtosis, and the mean of LAVI, E peak, e’ peak and E/e’ ratio. The solid and dotted lines represent the normal and kernel density distributions. The *P* value is for departure of the actually observed distribution from normality according to the Shapiro-Wilk test.


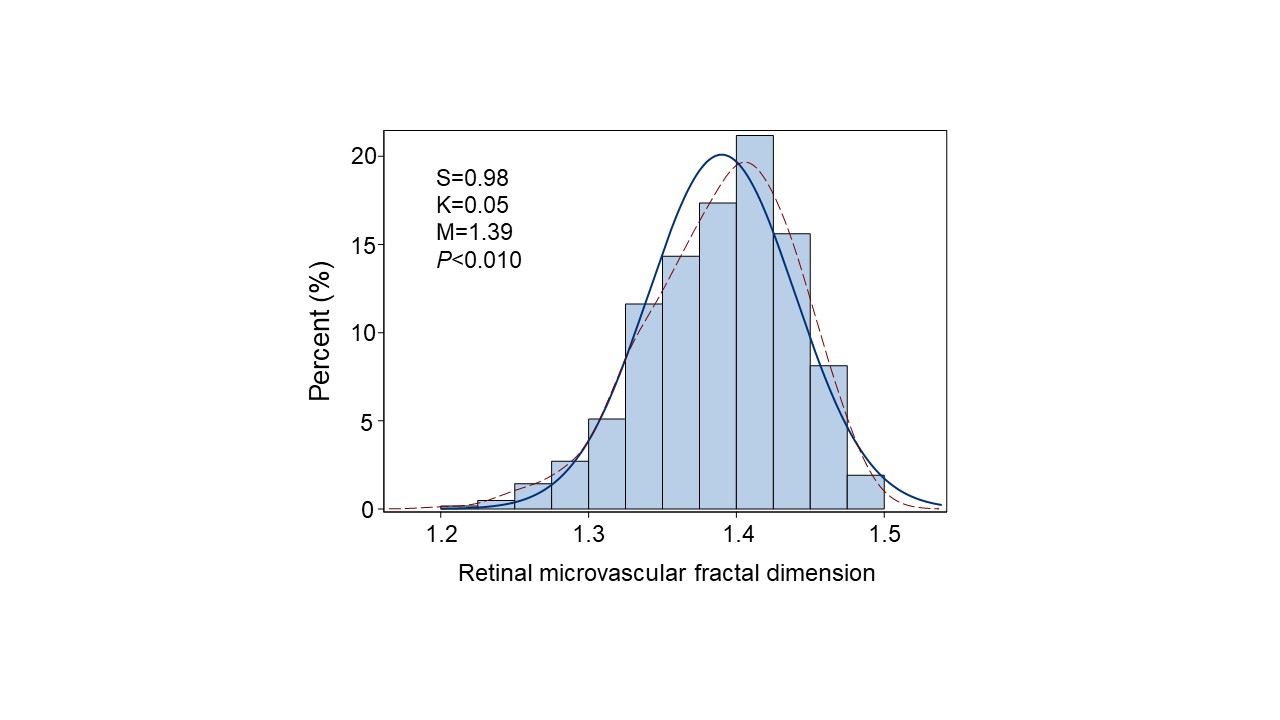


**Supplementary Figure 3**

Frequency distribution of retinal microvascular fractal dimension. S, K and M are the coefficients of skewness and kurtosis, and the mean of retinal microvascular fractal dimension. The solid and dotted lines represent the normal and kernel density distributions. The *P* value is for departure of the actually observed distribution from normality according to the Shapiro-Wilk test.


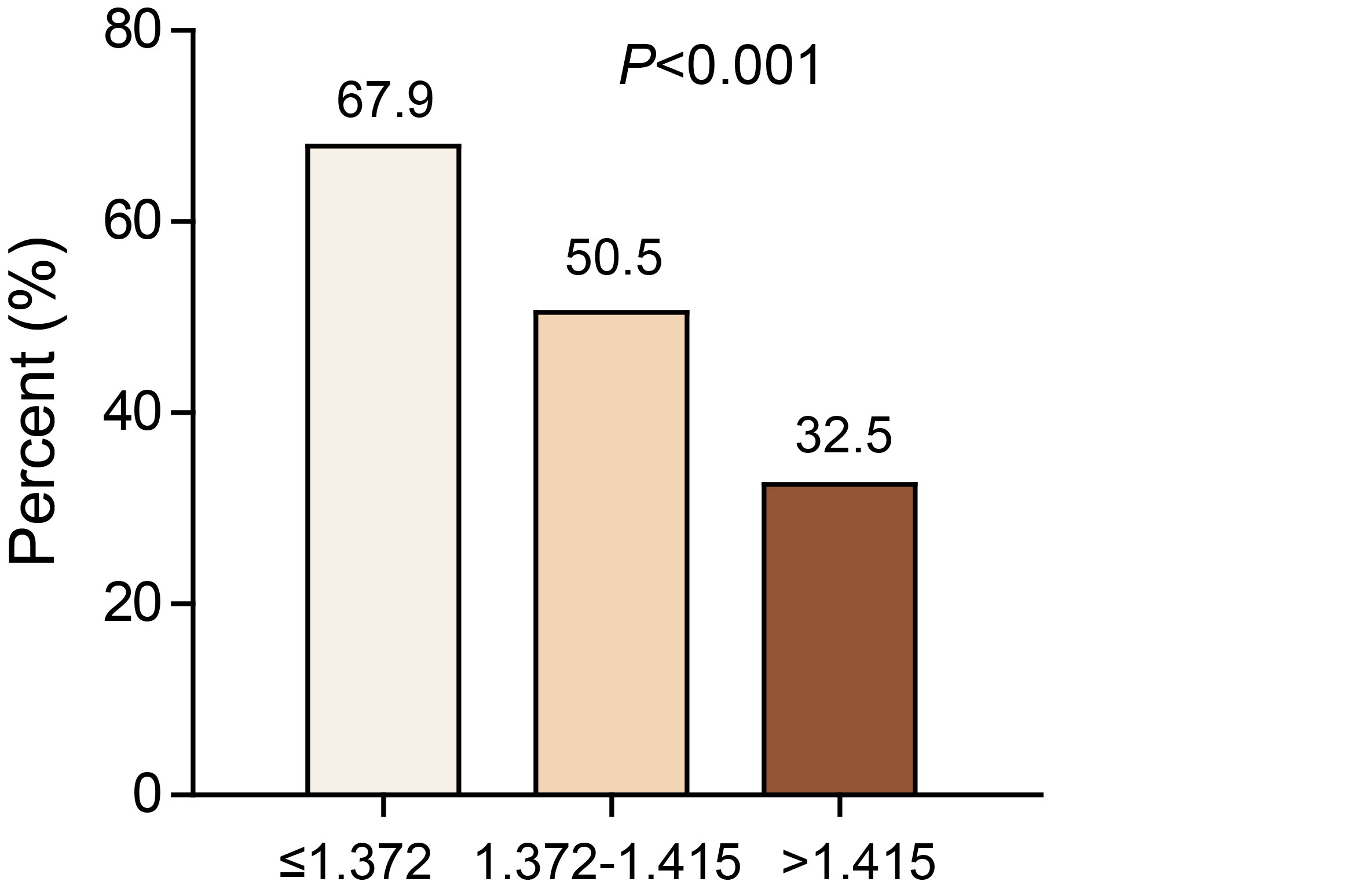


**Supplementary Figure 4**

Prevalence of low and high E/e’ ratio (≤6.5 and >6.5, respectively) by thirds of the distribution of retinal microvascular fractal dimension. The *P* value is for the trend.


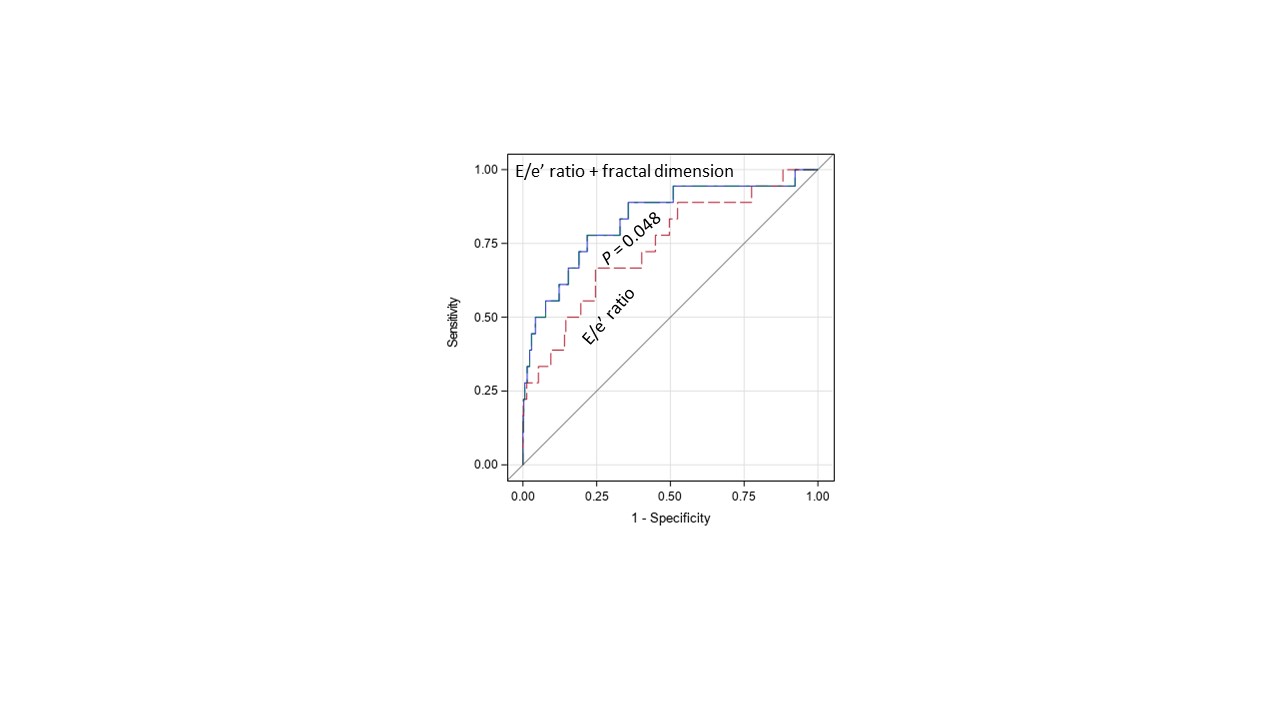


**Supplementary Figure 5**

Receiver operating characteristics plots discriminates the area under the curve between E/e’ ratio (red line) and E/e’ ratio plus retinal microvascular fractal dimension (blue line). Combining retinal microvascular fractal dimension with E/e’ ratio increased (*P* = 0.048) the area under the curve from 0.74 to 0.83.
